# Predicting Chemical Carcinogens Using a Hybrid Neural Network Deep Learning Method

**DOI:** 10.3390/s22218185

**Published:** 2022-10-26

**Authors:** Sarita Limbu, Sivanesan Dakshanamurthy

**Affiliations:** Lombardi Comprehensive Cancer Center, Georgetown University Medical Center, Washington, DC 20057, USA

**Keywords:** chemical carcinogens, machine learning, deep learning neural network, hybrid neural network, convolution neural network, fast forward neural network

## Abstract

Determining environmental chemical carcinogenicity is urgently needed as humans are increasingly exposed to these chemicals. In this study, we developed a hybrid neural network (HNN) method called HNN-Cancer to predict potential carcinogens of real-life chemicals. The HNN-Cancer included a new SMILES feature representation method by modifying our previous 3D array representation of 1D SMILES simulated by the convolutional neural network (CNN). We developed binary classification, multiclass classification, and regression models based on diverse non-congeneric chemicals. Along with the HNN-Cancer model, we developed models based on the random forest (RF), bootstrap aggregating (Bagging), and adaptive boosting (AdaBoost) methods for binary and multiclass classification. We developed regression models using HNN-Cancer, RF, support vector regressor (SVR), gradient boosting (GB), kernel ridge (KR), decision tree with AdaBoost (DT), KNeighbors (KN), and a consensus method. The performance of the models for all classifications was assessed using various statistical metrics. The accuracy of the HNN-Cancer, RF, and Bagging models were 74%, and their AUC was ~0.81 for binary classification models developed with 7994 chemicals. The sensitivity was 79.5% and the specificity was 67.3% for the HNN-Cancer, which outperforms the other methods. In the case of multiclass classification models with 1618 chemicals, we obtained the optimal accuracy of 70% with an AUC 0.7 for HNN-Cancer, RF, Bagging, and AdaBoost, respectively. In the case of regression models, the correlation coefficient (R) was around 0.62 for HNN-Cancer and RF higher than the SVM, GB, KR, DTBoost, and NN machine learning methods. Overall, the HNN-Cancer performed better for the majority of the known carcinogen experimental datasets. Further, the predictive performance of HNN-Cancer on diverse chemicals is comparable to the literature-reported models that included similar and less diverse molecules. Our HNN-Cancer could be used in identifying potentially carcinogenic chemicals for a wide variety of chemical classes.

## 1. Introduction

Substances capable of causing cancer are known as carcinogens. Carcinogenicity is a primary concern among all the toxicological endpoints due to the severity of its outcome. Carcinogens may be genotoxic, which induces DNA damage and cancer, or non-genotoxic, which uses other modes of action, such as tumor promotion, to exhibit their carcinogenic potential in humans [1]. Some of the genotoxic carcinogens are mutagens too. Many environmental chemicals have been identified as carcinogenic to humans [2,3]. The onset of cancer in humans depends on various factors, including the dose and duration of exposure to carcinogens. Identifying carcinogenic compounds is also an integral step during the drug development process. The two-year rodent carcinogenicity assay has been established as the standard to determine chemical carcinogenicity [4]. However, such animal testing is time-consuming, costly, and unethical. The experimentalists need to replace, reduce, and refine (3Rs) the use of animals as this 3Rs policy encourages alternative methods to minimize the unprincipled use of animals [5].

Computational methods for various toxicological endpoints prediction have now become a popular alternative to traditional animal testing. Numerous computational models using machine learning (ML) methods are developed to predict carcinogenicity based on the properties of chemicals. Computational models can be classification models (qualitative) that predict chemical is carcinogenic/noncarcinogenic (binary classification models) or that predict the degree of carcinogenicity (multiclass classification), and regression models (quantitative) that predict the dose of chemical required for carcinogenesis. Computational models based on structurally related congeneric chemicals are reported to have high predictive performance. Luan et al. reported an accuracy of 95.2% while predicting the carcinogenicity of N-nitroso compounds based on the support vector machine (SVM) method [6]. Ovidiu et al. presented a SVM-based model to predict the carcinogenicity of polycyclic aromatic hydrocarbons (PAH) with 87% accuracy [7]. Computational models based on non-congeneric chemicals are of interest due to their predictive ability for diverse chemicals. Fjodorova et al. predicted the carcinogenicity of non-congeneric chemicals with 68% accuracy using a counter propagation artificial neural network (CP ANN) [8]. Tanabe et al. reported an accuracy of 70% for non-congeneric chemicals based on SVM and improved the accuracy to 80% by developing models on the chemical subgroups based on their structure [9]. Zhang et al. presented binary classification models based on ensemble of the extreme gradient boosting (XGBoost) method that predicted the carcinogenicity of chemicals with 70% accuracy [10]. Li et al. used six different ML methods to generate the binary classification model with 83.91% accuracy and ternary (multiclass) classification models with 80.46% accuracy for the external validation set for the best model [11]. Toma et al. developed binary classification models with an accuracy of 76% and 74% and regression models with r^2^ of 0.57 and 0.65 on oral and inhalation slope factors to predict carcinogenicity for the external validation set [12]. Fjodorova et al. reported a correlation coefficient of 0.46 for the test set for their regression models using counter propagation artificial neural network (CP ANN) [8]. Wang et al. constructed a deep learning model that requires fewer data and achieved 85% accuracy on the external validation set for carcinogenicity prediction [13].

Taken together, numerous carcinogenicity predictive models on congeneric and non-congeneric chemicals for binary classification and a few multiclass and regression models were reported [6,7,8,9,10,11,12,13,14,15,16,17]. However, there is a need for more non-congeneric computational models with a broad applicability domain for carcinogenicity prediction. In this study, to predict potential carcinogens, we developed a hybrid neural network method called, HNN-Cancer. Based on diverse non-congeneric chemicals, we have developed binary classification, multiclass classification, and regression models, using HNN-Cancer and other machine learning methods. We have used the binary classification to predict a chemical is carcinogenic or non-carcinogenic, the multiclass classification model to predict the severity of the chemical carcinogenicity, and the regression model to predict the median toxic dose.

## 2. Materials & Methods

### 2.1. Datasets

We have collected carcinogens from several different data sources detailed below.

1. Chemical Exposure Guidelines for Deployed Military Personnel Version 1.3 (MEG).
  We curated carcinogenic chemicals from the Technical Guide 230 (TG230): “Chemical Exposure Guidelines for Deployed Military Personnel” [18]. TG 230 provides military exposure guidelines (MEGs) for chemicals in the air, water, and soil, along with an assigned carcinogenicity group for each chemical. Chemicals are categorized into one of 5 groups: Group A (human carcinogen), Group B (probable human carcinogen), Group C (possible human carcinogen), Group D (not classifiable), and Group E (no evidence of carcinogenicity).
2. Environmental Health Risk Assessment and Chemical Exposure Guidelines for Deployed Military Personnel 2013 Revision (TG230).
  We curated carcinogenic chemicals listed in the Technical Guide 230 (TG230): “Environmental Health Risk Assessment and Chemical Exposure Guidelines for Deployed Military Personnel” [19], which provides military exposure guidelines (MEGs).
3. National Toxicology Program (NTP).
  Carcinogenic chemicals were curated from the NTP [20]. NTP lists two groups of carcinogenic chemicals: (a) reasonably anticipated to be a human carcinogen and (b) known to be human carcinogens.
4. International Agency for Research on Cancer (IARC)
  Carcinogenic chemicals were curated from IARC [21]. IARC categorizes chemicals into one of the 5 groups: Group 1 (carcinogenic to humans), Group 2A (probably carcinogenic to humans), Group 2B (possibly carcinogenic to humans), Group 3 (not classifiable as to its carcinogenicity to humans), and Group 4 (probably not carcinogenic to humans).
5. The Japan Society for Occupational Health (JSOH)
  Carcinogenic chemicals were curated with the recommendation of Occupational Exposure Limits published by the JSOH [22], which are classified into one of the 3 groups: Group 1 (carcinogenic to humans), Group 2A (probably carcinogenic to humans), and Group 2B (possibly carcinogenic to humans).
6. The National Institute for Occupational Safety and Health (NIOSH)
  Carcinogenic chemicals curated from the NIOSH [23].
7. Carcinogenic Potency Database (CPDB)
  - CPDB_CPE (CPDB CarcinoPred-EL) data: CPDB data for rat carcinogenicity were collected from the CarcinoPred-EL developed by Zhang et al. [10]. The list contains 494 carcinogenic and 509 non-carcinogenic chemicals.
  - CPDB data: CPDB [24] data were collected and processed to obtain the median toxicity dose (TD50) for rat carcinogenicity. TD50 is the dose-rate in mg/kg body wt/day administered throughout life that induces cancer in half of the test animals. A total of 561 carcinogenic chemicals was obtained with TD50 values for rat carcinogenicity. A total of 605 noncarcinogenic chemicals was obtained for rat carcinogenicity. For 543 carcinogenic chemicals out of 561, the TD50 values in mmol/kg body wt/day were also obtained from the DSSTox database (https://www.epa.gov/chemical-research/distributed-structure-searchable-toxicity-dsstox-database; accessed on 30 September 2017).
8. Chemical Carcinogenesis Research Information System (CCRIS).
  Carcinogenesis data were collected from the CCRIS at ftp://ftp.nlm.nih.gov/nlmdata/.ccrislease/; accessed on 30 September 2017. The carcinogenicity and mutagenicity data were extracted. A total of 6833 chemicals was obtained after eliminating duplicates/conflicting data when compared to data sources 1 to 6, out of which 4054 were carcinogenic/mutagenic and 2779 were non-carcinogenic/mutagenic.
9. Drugbank 2018
  The drug data were collected from the drug bank (www.drugbank.ca; accessed on 31 March 2018). The approved drugs predicted as carcinogenic by Zhang et al. [10] were removed, the remining 1756 approved drugs were considered non-carcinogenic.

#### 2.1.1. Dataset I: Binary Classification Data

The two classes considered in the binary classification models were class 0 (non-carcinogen) and class 1 (carcinogen). Datasets used to train the models are listed below:

- For binary classification of chemicals to predict the carcinogenic or non-carcinogenic category, 448 carcinogenic chemicals were obtained from data sources 1 to 6 above.
  Data 1 (MEG): The chemicals classified into Groups A, B, and C were considered as carcinogens.
  Data 2 (TG30): The chemicals listed as carcinogens were considered as carcinogens.
  Data 3 (NTP): The chemicals classified as either “reasonably anticipated to be a human carcinogen” or “known to be human carcinogens” were considered as carcinogens.
  Data 4 (IARC): The chemicals classified into Groups 1, 2A, and 2B were considered as carcinogens.
  Data 5 (JSOH): The chemicals classified into Groups 1, 2A, and 2B were considered as carcinogens.
  Data 6 (NIOSH): The carcinogenic chemicals listed were considered as carcinogens.
- CPDB_CPE chemicals from data source 7a contributed 320 carcinogenic and 458 non- carcinogenic additional data after comparing to the data from data sources 1 to 6 and removing duplicates and conflicting chemicals.
- The CCRIS mutagenicity/carcinogenicity data from data source 8 contributed 3868 mutagenic/carcinogenic data and 2500 non-mutagenic/carcinogenic data.
- A total of 400 non-carcinogenic approved drugs from data source nine was also used in this classification model.

For the binary classification model dataset, we used 7994 chemicals with 4636 carcinogenic and 3358 non-carcinogenic chemicals.

#### 2.1.2. Dataset II: Multiclass Classification Data

The classes considered in the multiclass classification models were class 0 (non-carcinogen), 1 (possibly carcinogen and not classifiable chemicals), and 2 (carcinogen and probably carcinogen). Datasets used to train the models are listed below:

- For multiclass classification, 882 carcinogenic and 2 non-carcinogenic chemicals were collected from data sources 1, 3, 4, and 5. There was a total of 2 in class 0, 604 in class 1, and 278 in class 2 in this dataset.
  Data 1 (MEG): The chemicals classified into Groups A and B were considered class 2. The chemicals classified into Groups C and D were considered class 1 carcinogens. Chemicals classified into group E are considered class 0 compounds.
  Data 3 (NTP): The chemicals classified as either “reasonably anticipated to be a human carcinogen” or “known to be human carcinogens” were considered class 2.
  Data 4 (IARC): The chemicals classified into Groups 1 and 2A were considered class 2 carcinogens, and those classified into Groups 2, B, and 3 were considered class 1 carcinogens.
  Data 5 (JSOH): The chemicals classified into Groups 1 and 2A were considered class 2 carcinogens, and those classified into Groups 2B were considered class 1 carcinogens.
  Considering Group D of MEG data as class 1 carcinogen along with Group C and considering Group 3 of IARC data as class 1 carcinogen along with Group 2B increased the multiclass data significantly in this dataset. In the case of binary classification, we discarded these groups.
- CPDB chemicals from data source 7b contributed 277 carcinogenic and 457 non-carcinogenic additional data after removing duplicates and conflicting chemicals compared to the data from data sources 1, 3, 4, and 5. The 277 carcinogenic chemicals were categorized into class 2, and 457 noncarcinogenic chemicals were categorized into class 0.

The dataset II for the multiclass classification models, we used a total of 459 chemicals data in class 0, 604 chemicals data in class 1, and 555 chemicals data in class 2.

#### 2.1.3. Dataset III: Regression Data

Regression models were developed to predict the quantitative carcinogenicity or the median toxic dose (TD50) of the chemicals in the form of pTD50 (logarithm of the inverse of TD50). Dataset III for the regression models consisted of 561 TD50 data in mg/kg body wt/day converted to pTD50 from data source 7b. Independently, the regression models were also developed on 543 TD50 data in mmol/kg body wt/day converted to pTD50.

### 2.2. Descriptors

Mordred descriptor calculator [25] that calculates 1613 2D molecular descriptors from SMILES and is used for descriptor calculation. This descriptor calculator supports Python 3 that we used to run the Mordred locally. The final set of 653 descriptors was obtained with no missing calculated values for the entire datasets for which descriptors were calculated. The 653 descriptors were used as a final set of input features for the training and test data set for the machine learning models.

### 2.3. SMILES Preprocessing

The simplified molecular-input line-entry system (SMILES) uses ASCII strings for the 1D chemical structure representation of a compound and can be used to convert to its 2-D or 3-D representation. It is one of the key chemical attributes and is used in our deep learning model. Raw texts cannot be directly used as input for the deep learning models but should be encoded as numbers. Tokenizer class in python is used to encode the SMILES string. The SMILES preprocessing method that we used while predicting toxicity [17] created the index for the set of unique characters of SMILES from the training set only. If the training set consists of only two compounds “C=CC=C” and “O=CC, a dictionary would be created for only three distinct characters in the SMILES of the training set that would map C to 1, = to 2, and O to 3. Then, the vector output for the SMILES characters was one-hot encoded where the categorical value of each character in the SMILES is converted to binary vector with only the index set to 1. Thus, C, =, and O are represented by the vectors [1 0 0], [0 1 0], and [0 0 1], respectively. If a new character, such as ‘N’, which does not exist in the training set, appears in the SMILES of the test set, the character would be skipped. For the string C=CC#N, the SMILES vectorization method would output the following matrix of dimension LxM, where L = 325 is the allowed maximum length of the SMILES string and M is the number of the unique characters in the SMILES of the training set:

$$\left[\begin{array}{c} C\\ =\\ C\\ C\\ \#\\ N \end{array}\right]=\left[\begin{array}{ccc} 1 & 0 & 0\\ 0 & 1 & 0\\ 1 & 0 & 0\\ 1 & 0 & 0\\ 0 & 0 & 0\\ \vdots & \vdots & \vdots \\ 0 & 0 & 0 \end{array}\right]$$

Here, in the modified vectorization method, we have created a unique index for 94 characters in the ASCII table. Hence, there is no possibility of missing out on creating an index of any character in the SMILES string represented in any format. A total of 94 characters in the ASCII table !, “, #, …, =, >, ?, @, A, B, C, …, |, }, ~ represented by decimal numbers 33, 34, 35, …, 61, 62, 63, 64, 65, 66, 67, … 124, 125, 126, respectively, made the vocabulary of the possible characters in the SMILES. Each of these 94 ASCII characters were obtained by looping through the numbers 33 through 126 and converting the number to the corresponding character using python function chr(). Then, the characters were mapped to indices 1, 2, 3, …, 29, 30, 31, 32, 33, 34, 35, …, 92, 93, 94 using the fit_on_texts() function of the Tokenizer module to create a dictionary.

Each character in the SMILES is converted to its corresponding index in the dictionary, and a vector is created for the SMILES of each compound. As an example, acrylonitrile-d3 with SMILES string C=CC#N is encoded as [35, 29, 35, 35, 3, 46]. As the SMILES length varies depending on the compound’s length and properties, the length of the encoding results also varies. The resulting vector for the SMILES of every input compound is thus padded with 0s or truncated so that they are of uniform length, L. The SMILES for the input compounds are converted to a 2-D matrix of size K x L, where K is the number of input SMILES, and L = 325 is the allowed maximum length of the SMILES string used in the model. Thus, for the string C=CC#N, the current SMILES vectorization method would output the following vector of length 325:

[35, 29, 35, 35, 3, 46, 0, 0, …, 0]

Our previous method [17] mapped the SMILES for the K number of chemicals to a one-hot encoded matrix of size KxLxM, where M is the number of the possible characters in the SMILES.

## 3. Machine Learning Models

### 3.1. Hybrid Neural Network Model

Hybrid neural network (HNN) model [17] that we developed for chemical toxicity prediction was used here by modifying the SMILES vectorization method. Then, the method by which the vectorized SMILES input is processed by the convolutional neural network (CNN) of the model. The model is developed in python using the Keras API with Tensorflow in the backend. The model consists of a CNN for deep learning based on structure attribute (SMILES) and a multilayer perceptron (MLP)-type feed-forward neural network (FFNN) for learning based on descriptors of the chemicals. To vectorize SMILES, each character in the SMILES string is converted to its positional index in the dictionary, as explained in the SMILES preprocessing section. The 2D array of vectorized SMILES strings was the input for the CNN. The embedding layer of Keras is used to convert the index of each character in the SMILES string into a dense vector. The embedding layer takes three arguments as input: input_dim is the vocabulary size of the characters in the SMILES string, output_dim is the size of the embedded output for each character, and input_length is the length of the SMILES string. In the model, we have embedded the index of each character in the SMILES to a vector of size 100 by setting the output_dim to 100. The embedding layer converts the input 2D array of size KxL, where K is the number of SMILES and L is the maximum length of SMILES, to a 3D array of size KxLx100.

The 1D convolution layer activation function ReLU represented mathematically as max(0, x), is used in the model that replaces all the negative values with zeros. The derivative of ReLU is always 1 for positive input, which counteracts the vanishing gradient problem during the backpropagation. The output of the pooling layer of the CNN, together with the FFNN, is connected to the final fully connected layer to perform the classification task.

### 3.2. Other Machine Learning Algorithms

To test the performance of HNN-Cancer for the case of binary classification and multiclass classification, the other machine learning algorithms random forest (RF), bootstrap aggregating (Bagging) using bagged decision tree, and adaptive boosting (AdaBoost), were used.

Random forest (RF): A bootstrap aggregating (bagging) model that uses ensemble decision trees to make final decisions. This algorithm uses only a subset of features to find the best feature to separate classes at each node of the tree. The regression model fits every feature, and the data are split at several points. The feature with the least error is selected as the node.

Bagged decision tree (Bagging): Bagging uses a bootstrap method to reduce variance and overfitting. It uses the ensemble method for the final decision. Bagging method uses all features to find the best feature for the splitting node of the tree.

Adaptive boosting (AdaBoost): AdaBoost is an ensemble machine learning method that uses weak classifiers to make stronger classifiers.

Support vector regressor (SVR): SVR depends on the subset of training data. SVR performs non-linear regression using kernel trick and transforms inputs into m-dimensional feature space.

Gradient boosting (GB): GB produces an ensemble of weak prediction models or regression trees in a stage-wise fashion. Each stage optimizes a loss function by choosing the function that points in the negative gradient direction.

Kernel ridge (KR): Ridge regression uses L2 regularization to limit the size of the coefficients of the model and eliminates the problem in the least square regression. The ridge method adds a penalty to the coefficients equal to the square of the magnitude of coefficients. Regularization parameter λ controls the penalty term. Kernel ridge uses kernel tricks to make the model non-linear.

Decision tree with AdaBoost (DT): The prediction of the decision tree was boosted with AdaBoost. The decision tree method predicts by learning decision rules from the training data. AdaBoost is a boosting algorithm introduced by Freund and Schapire [26]. AdaBoost makes final predictions from weighted voting of the individual predictions from weak learners. It implements AdaBoost.R2 algorithm [27].

KNeighbors (KN): Nearest neighbors find k number of training data closest to the test data for which prediction is made. Each closest neighbor contributes equally while making a prediction (default parameter).

### 3.3. Model Evaluation

All the statistical metric results presented for the model evaluation are the average of 10 repeats (in the case of binary classification models and regression models) and 30 repeats (in the case of multiclass classification models). Approximately 20% of data were separated randomly in each iteration as test sets and the remaining data as training sets, such as five-fold cross-validation, except that the test sets were randomly selected in each iteration. In the case of binary and multiclass classification, the performance of each model was evaluated based on accuracy and area under the receiver operating characteristic curve (AUC). The classification models were also assessed for sensitivity and specificity. The evaluation scores are calculated as:

$$Accuracy=\frac{TP+TN}{TP+TN+FN+FP}\times 100$$

$$Sensitivity\;\left(TPR\right)=\frac{TP}{TP+FN}\times 100$$

$$Specificity\;\left(TNR\right)=\frac{TN}{TN+FP}\times 100$$

For the five-fold cross-validation, we used 80:20 training to test set ratios, which are good numbers for the significant data size used in this study. Further, the data are shuffled in each iteration before separating the training and the test set to make sure the process does not end up with a dataset containing bias in both the training and the test set. Additionally, the average performance metrics were calculated from the outcome of 10 simulations in the case of binary classification models and regression models. Whereas for the multiclass classification models, the average performance metrics were calculated from the outcome of 30 simulations. The training on 80% of the data give more room for better performance (compared to 10-fold cross-validation with 90% data in the training set) while predicting for an external dataset using a model trained on 100% of the data.

In the multiclass classification, micro averaging is used to obtain the average of the metrics of all the classes. Micro averaging involves calculating the average by converting the data in multiple classes to binary classes and giving equal weight to each observation. In multiclass classification with the imbalanced dataset, micro averaging of any metric is preferred when compared to macro averaging, which involves calculating the metrics separately for each class and then averaging them by giving equal weight to each class. In the case of multiclass classification with *n* number of classes,

$$Acc_{micro}=\frac{\left(TP_{1}+TP_{2}+\cdots +TP_{n}\right)+\left(TN_{1}+TN_{2}+\cdots +TN_{n}\right)}{\left(TP_{1}+\cdots +TP_{n})+(TN_{1}+\cdots +TN_{n})+(FN_{1}+\cdots +FN_{n})+(FP_{1}+\cdots +FP_{n}\right)}$$

$$Sensitivity_{micro}=\frac{TP_{1}+TP_{2}+\cdots +TP_{n}}{\left(TP_{1}+TP_{2}+\cdots +TP_{n})+(FN_{1}+FN_{2}+\cdots +FN_{n}\right)}\times 100$$

$$Specificity_{micro}=\frac{TN_{1}+TN_{2}+\cdots +TN_{n}}{\left(TN_{1}+TN_{2}+\cdots +TN_{n})+(FP_{1}+FP_{2}+\cdots +FP_{n}\right)}\times 100$$

where *TP* = true positive, *TN* = true negative, *FP* = false positive, *FN* = false negative, *TPR* = true positive rate, *TNR* = true negative rate.

The performance of each regression model was evaluated based on the coefficient of determination (R^2^). The coefficient of determination gives the percentage of variation in the dependent variable that is predictable from the independent variable, or that is explained by the independent variable.

(1) $$R^{2}=\frac{ESS}{TSS}=\frac{\sum_{i=1}^{n}{\left({\hat{y}}_{i}-\bar{y}\right)}^{2}}{\sum_{i=1}^{n}{\left(y_{i}-\bar{y}\right)}^{2}}$$

where *ESS* is explained as the sum of squares, and *TSS* is the total sum of squares; ${\hat{y}}_{i}$ is the predicted value of the *i*th dependent variable; $y_{i}$ is the *i*th observed dependent variable; and $\bar{y}$ is the mean of the observed data.

## 4. Results and Discussion

It is a desperate need to efficiently evaluate potential carcinogenic compounds that humans are exposed to in preventing cancer incidence, progression, and high mortality. Several computational and machine learning models have been developed for the prediction of carcinogenic compounds [6,7,8,9,10,11,12,13,14,15,16,28,29,30,31,32,33,34,35,36,37,38,39,40]. However, most or all of the models are developed as binary or regression models, not as categorical multiclassification models or comprehensive classification models. Further, these models are limited to congeneric computational models with a limited applicability domain and small dataset; they lack chemical diversity and were applied to targeted organ systems for carcinogenicity prediction. To fill this gap, we developed HNN-Cancer, a deep learning-based hybrid neural network model and predicted the carcinogenicity in large scale with a variety of datasets. The HNN-Cancer combines two neural network models, the CNN and the FFNN. The HNN-Cancer model combines CNN for deep learning based on the structure attribute (SMILES) with a multilayer perceptron (MLP)-type feed-forward neural network (FFNN) for learning based on descriptors of the chemicals. We developed different classification models, such as binary classification, multiclass classification, and regression models based on diverse non-congeneric chemicals.

The HNN carcinogenicity prediction models are developed based on the hybrid neural network (HNN) architecture we reported previously for toxicity prediction [17]. To compare the HNN prediction performance, we also developed other machine learning models, such as random forest (RF), bootstrap aggregating (Bagging), and adaptive boosting (AdaBoost) for binary classification and multiclass classification. Several regression models were developed based on random forest (RF), support vector regressor (SVR), gradient boosting (GB), kernel ridge (KR), decision tree with AdaBoost (DT), and KNeighbors (KN) using the sklearn package in python to make the final consensus prediction of the median toxic dose (TD50). A consensus prediction was calculated based on the average of all seven predicted values. We used the modified version of the 3D array representation of 1D SMILES in the convolutional neural network (CNN) in the HNN models from our previous model [17]. The SMILES processing method included a vocabulary of 94 characters in the ASCII table so as not to miss any possible characters of SMILES in any format. Additionally, instead of using one-hot encoding to vectorize the characters in the 1-D SMILES, the embedding layer of the CNN was used.

### 4.1. Carcinogen Prediction Using Binary Classification

**Figure 1:**
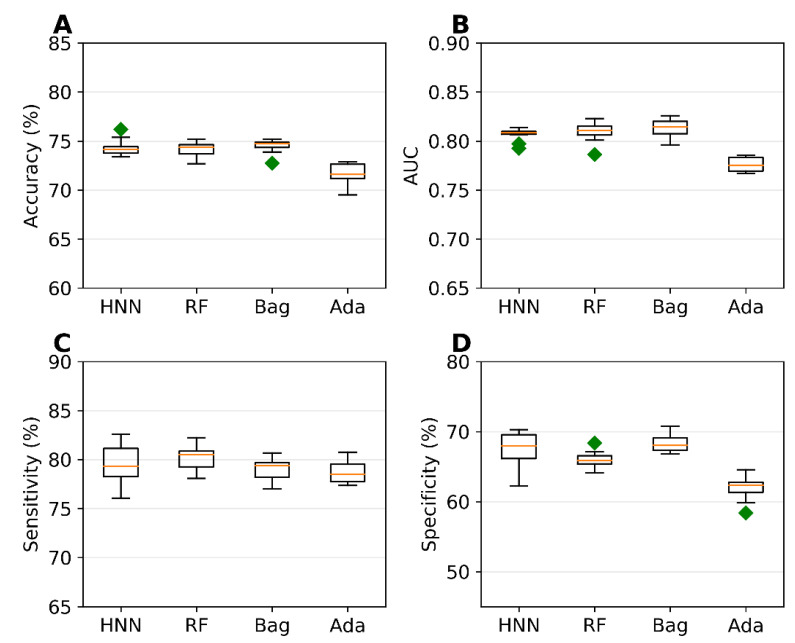
(**A**) accuracy, (**B**) AUC, (**C**) sensitivity, and (**D**) specificity for the dataset I as given by the binary classification models developed based on the HNN, RF, Bagging, and AdaBoost methods.

The binary classification models were developed for Dataset I comprising 7994 chemicals (4636 carcinogenic and 3358 noncarcinogenic) from 9 different sources. Out of 1613 descriptors calculated by the Mordred descriptor calculator, 653 descriptors with no missing values were used to develop the models. We used the SMILES string in addition to the 653 descriptors in the HNN model. The accuracy, AUC, sensitivity, and specificity of the HNN-Cancer, RF, and Bagging models were comparable, whereas AdaBoost statistical metrics were significantly lower (Figure 1). The accuracy of the three models was 74%, and their AUC was ~0.81. The sensitivity and specificity of the HNN model was 79.47% and 67.3%.

Zhang et al. [10] built several machine learning models on the CPDB’s 1003 carcinogenic data on rats. The highest accuracy they reported was 70.1%, and an AUC of 0.765 for the five-fold cross-validation. Wang et al. [13] developed a deep learning tool CapsCarcino on the 1003 rat data from CPDB used by Zhang et al. For five-fold cross-validation, they reported accuracy of 74.5%, a sensitivity of 75%, and specificity of 74.2%. Li et al. developed 30 models on only 829 rat data from CPDB, with the highest accuracy of 89.29% on their test set. Tanabe et al. [9] developed an SVM model with an accuracy of 68.8% and an AUC of 0.683 for non-congeneric chemicals from six sources using dual cross-validation. They improved the accuracy by developing models on congeneric subgroups. Notably, these studies clearly demonstrate that models developed on more diverse chemicals result in reduced accuracy. In contrast, the predictive performance of our HNN-Cancer models based on a highly diverse set of chemicals is still good compared to the previously reported models with a high AUC. Hence, we expect the HNN-Cancer will rapidly make optimal carcinogen predictions for a wider variety of chemicals.

### 4.2. Carcinogen Prediction Using Multiclass Classification

**Figure 2:**
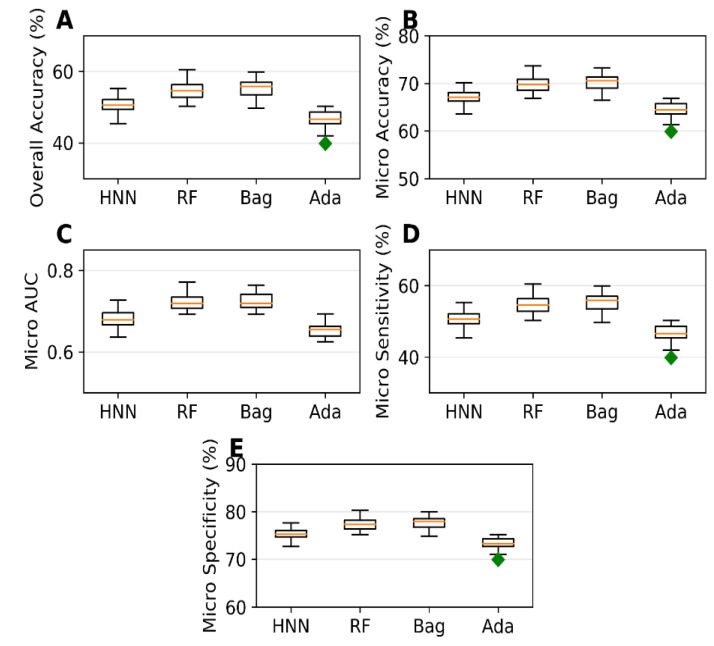
(**A**) Overall accuracy, (**B**) micro accuracy, (**C**) micro AUC, (**D**) micro sensitivity, and (**E**) micro specificity for the dataset II as given by the multiclass classification models developed based on HNN, RF, Bagging, and AdaBoost methods.

The multiclass classification models were developed for Dataset II, containing 1618 chemicals with 459 chemicals in class 0, 604 in class 1, and 555 in class 2. In contrast, class 0 comprises non-carcinogens, class 1 comprises possible carcinogens and not classifiable chemicals, and class 2 comprises carcinogens and probable carcinogens. The overall accuracy is 50.58%, 54.73%, 55.52%, and 46.50%, the micro accuracy is 67.05%, 69.82%, 70.34%, and 64.33% whereas the average micro AUC is 0.68, 0.724, 0.725, and 0.653 for HNN-Cancer, RF, Bagging, and AdaBoost, respectively (Figure 2). As observed by Limbu et al. [17], the HNN-Cancer model is not performing better for the multiclass in comparison to RF and Bagging method. This is because the deep learning method performs best with a large dataset, and the dataset used in these two studies is not sufficiently large.

Li et al. developed 30 multiclass (ternary) classification models that categorized compounds into carcinogenic I (strongly carcinogenic), carcinogenic II (weakly carcinogenic), and non-carcinogens [11]. Their kNN model based on MACCS fingerprint with the best predictive performance achieved micro accuracy of 81.89%. The ternary classification of their data was based on the TD50 values where TD50 ≤ 10 mg/kg/day were carcinogenic I and TD50 > 10 mg/kg/day were carcinogenic II. Whereas the classification of data in our models is based on their category, they are class 2 if they are carcinogenic or probably carcinogenic, class 1 if they are possibly carcinogenic or not classifiable chemicals, class 0 if they are non-carcinogenic. All the data from CPDB with TD50 were classified as class 2, and non-carcinogens were classified as class 0; yet, none of them classified as class 1. However, we provided a complete classification range coverage when predicting the chemical carcinogenicity.

### 4.3. Carcinogenicity Prediction Using Regression

**Figure 3:**
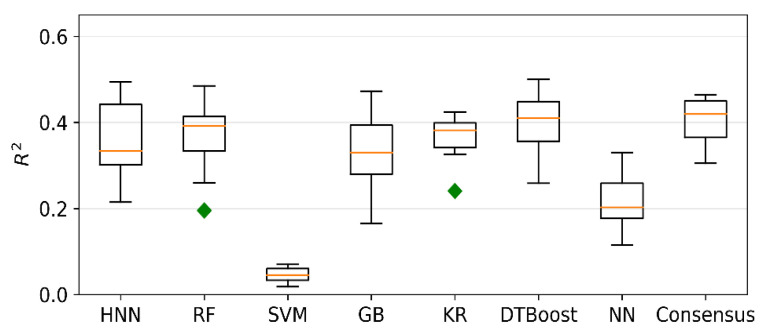
R^2^ of regression models developed based on HNN-Cancer, RF, SVM, GB, KR, DTBoost, NN, and consensus methods.

**Figure 4:**
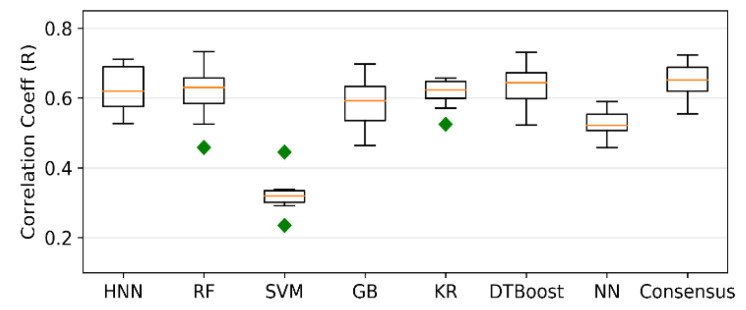
Correlation coefficient (R) of regression models developed based on HNN-Cancer, RF, SVM, GB, KR, DTBoost, NN, and consensus methods.

**Figure 5:**
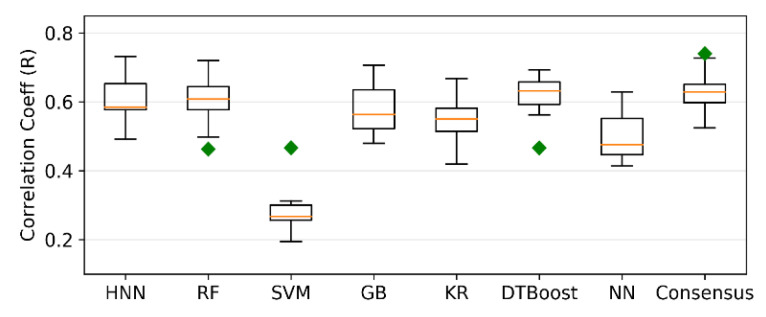
Correlation coefficient (R) of regression models developed based on HNN-Cancer, RF, SVM, GB, KR, DTBoost, NN, and consensus methods that predicts the carcinogenicity in mmol/kg body wt/day.

Regression models were developed for Dataset III comprising 561 TD50 chemicals. The models predicted carcinogenicity in the form of pTD50 (logarithm of the inverse of TD50), and the average of all seven predicted values was calculated as the final consensus prediction of the pTD50 value. The R^2^ is 0.35, 0.36, 0.04, 0.33, 0.36, 0.39, and 0.21 for the HNN-Cancer, RF, SVM, GB, KR, DTBoost, and NN methods, respectively (Figure 3). The overall R^2^ was slightly increased to 0.40 by the consensus prediction. The correlation coefficient (R) is 0.628, 0.611, 0.322, 0.588, 0.614, 0.636, 0.527, and 0.649 for the HNN, RF, SVM, GB, KR, DTBoost, NN, and consensus methods, respectively (Figure 4). The models were also developed for 543 TD50 data in mmol/kg body wt/day. The correlation coefficient (R) is 0.604, 0.601, 0.287, 0.577, 0.545, 0.617, 0.497, and 0.629 for the HNN-Cancer, RF, SVM, GB, KR, DTBoost, NN, and consensus methods, respectively (Figure 5).

Fjodorova et al. [8] developed the quantitative models for carcinogenicity prediction on 805 rat data from CPDB using counter propagation artificial neural network (CP ANN) [8]. The correlation coefficient of the models was 0.46 for the test set. Toma et al. developed regression models to predict the carcinogenicity for external validation set with r^2^ of 0.57 and 0.65 for models using oral and inhalation slope factor [12]. In the Toma et al. [12] study, only 315 out of 1110 oral and 263 out of 990 inhalation compounds were included in their final dataset after selecting compounds based on various criteria. The external validation set was randomly chosen from the finally obtained dataset with highly similar compounds. This may be the reason for a slightly better coefficient of determination reported by Toma et al. [12] compared to our models. Singh et al. [41] developed regression models based on generalized regression neural network (GRNN) to predict the carcinogenicity in mmol/kg body wt/day for 457 CPDB compounds and reported a correlation coefficient of 0.896 [41]. The high value of the correlation coefficient in comparison to our models could be attributed to the nine molecular descriptors selected for the regression models, and the GRNN method was used. Taken together, our model included the multiclassification models with full classification range coverage with diverse class of chemicals and provided optimal carcinogen predictive performance over the other methods.

## 5. Conclusions

Determining environmental chemical carcinogenicity is an urgent need. Though several machine learning models have been reported, there is a need for more non-congeneric computational models with a vast applicability domain for carcinogenicity prediction. In this study, we determined the carcinogenicity of thousands of wide-variety classes of real-life exposure chemicals. We have developed carcinogen prediction models based on our hybrid neural network (HNN) architecture method HNN-Cancer to determine chemical carcinogens. In the HNN-Cancer, we included new SMILES feature representation method. Using the HNN-Cancer and other machine learning methods, we predicted the carcinogen in terms of binary classification, multiclass classification, and regression models for the very diverse non-congeneric chemicals. Notably, the binary and multiclass classification models developed for the larger set of diverse chemicals were from diverse sources, most of which are human exposure-relevant chemicals.

The models based on the HNN-Cancer, RF, and Bagging methods predicted the carcinogens with an accuracy of 74% and an AUC of 0.81, which shows that the carcinogen predictions made by these models can be considered as optimal. Multiclass classification models to categorize the carcinogenicity of chemicals into one of the three classes: non-carcinogens, possible carcinogens/not classifiable chemicals, or carcinogens/probable carcinogens, were developed. The HNN-Cancer exhibited an accuracy of 50.58%, a micro accuracy of 67.05%, and a micro AUC of 0.68. Further, we developed regression models to predict the median toxic dose of chemicals in the form of pTD50. The consensus prediction achieved the overall R^2^ of 0.40 by calculating the average of all the methods. Though our model included very diverse chemical categories and a larger number of chemicals from different data sources, still our models could be able to predict the binary, categorical (multiclass), and quantitative (regression) carcinogenicity comparable to the other literature reported models that included smaller and similar chemicals. Therefore, our HNN-Cancer can be used to identify the potential carcinogens for any chemical.

Several studies described the design of IoT-enabled environmental pollution and toxicology using the artificial intelligence technique to improve human health [42,43,44,45,46,47]. For example, Aisha et al. [42] proposed a neural network model that includes IoT-based sensor to sense eight pollutants and report the status of air quality in real-time by using a cloud server and informing the presence of hazardous pollutants levels in the air. Shukla et al. [46] and Memon et al. [47] employed artificial intelligence neural network IoT-enabled big data pipeline to the identification of breast cancer. Similarly, the HNN-Cancer could be integrated into the IoT-enabled sensors to inform the presence of carcinogens.

## 6. Limitations

The developed hybrid neural network method HNN-Cancer is first in class with developing various classification models, such as binary classification, multiclass classification, and regression models based on diverse non-congeneric chemicals. These models would enable the scientific community to classify chemicals carcinogenicity at specific doses or dose ranges. However, there are some potential limitations that exist in the prediction of carcinogens. Firstly, lack of a large dose-dependent chronic in vitro and in vivo carcinogen dataset to train the model. Secondly, the HNN-Cancer method needs several routines of optimization with further refinement. We will improve HNN-Cancer method carcinogen predictions further by including more experimentally determined carcinogenic dose data (in vitro and in vivo) that we obtained recently from the National Toxicology Program (NTP), bioinformatics and toxicology group.

## Data Availability

Not applicable.
